# Metabolic phenotype of clinical and environmental *Mycobacterium avium* subsp. *hominissuis* isolates

**DOI:** 10.7717/peerj.2833

**Published:** 2017-01-03

**Authors:** Andrea Sanchini, Flavia Dematheis, Torsten Semmler, Astrid Lewin

**Affiliations:** 1 Division 16, Mycotic and Parasitic Agents and Mycobacteria, Robert Koch Institute, Berlin, Germany; 2 Institute of Microbiology and Epizootics, Free University Berlin, Berlin, Germany; 3 NG 1 Microbial Genomics, Robert Koch Institute, Berlin, Germany

**Keywords:** *Mycobacterium avium*, Biolog, OmniLog®, Metabolism, Phenotype microarray

## Abstract

**Background:**

*Mycobacterium avium* subsp. *hominissuis* (MAH) is an emerging opportunistic human pathogen. It can cause pulmonary infections, lymphadenitis and disseminated infections in immuno-compromised patients. In addition, MAH is widespread in the environment, since it has been isolated from water, soil or dust. In recent years, knowledge on MAH at the molecular level has increased substantially. In contrast, knowledge of the MAH metabolic phenotypes remains limited.

**Methods:**

In this study, for the first time we analyzed the metabolic substrate utilization of ten MAH isolates, five from a clinical source and five from an environmental source. We used BIOLOG Phenotype Microarray^TM^ technology for the analysis. This technology permits the rapid and global analysis of metabolic phenotypes.

**Results:**

The ten MAH isolates tested showed different metabolic patterns pointing to high intra-species diversity. Our MAH isolates preferred to use fatty acids such as Tween, caproic, butyric and propionic acid as a carbon source, and L-cysteine as a nitrogen source. Environmental MAH isolates resulted in being more metabolically active than clinical isolates, since the former metabolized more strongly butyric acid (*p* = 0.0209) and propionic acid (*p* = 0.00307).

**Discussion:**

Our study provides new insight into the metabolism of MAH. Understanding how bacteria utilize substrates during infection might help the developing of strategies to fight such infections.

## Introduction

*Mycobacterium avium* subsp. *hominissuis* (MAH) is clinically one of the most relevant non-tuberculous mycobacteria (Tortoli, 2014). MAH is an opportunistic human pathogen causing pulmonary infections, lymphadenitis in small children and disseminated infections (Despierres et al., 2012; Rindi & Garzelli, 2014). It is of increasing public health relevance, with reports of MAH infections increasing worldwide (Hoefsloot et al., 2013). Moreover, MAH is widespread in the environment (Falkinham , 2013; Lahiri et al., 2014). In recent years, there have been substantial advances in the analysis of bacteria at the molecular level. Indeed, several whole genome sequences are now available for many mycobacterial species, including MAH (Bannantine et al., 2014; Kim et al., 2012; Uchiya et al., 2013; Wynne et al., 2010). In contrast, there has been little concomitant advance in knowledge at the phenotypic level. Phenotype analysis deserves greater attention, as it is the phenotype that selection pressure acts upon to confer evolutionary advantages to the bacterial species (Plata, Henry & Vitkup, 2015). In order to address this knowledge gap for bacterial phenotypes, BIOLOG Inc. developed the Phenotype MicroArray™ (PM) (BIOLOG, Hayward CA), a high throughput method for the rapid and global analysis of microbial metabolic phenotypes (Bochner, 2003; Bochner, 2009; Bochner, Gadzinski & Panomitros, 2001; Bochner, Giovannetti & Viti, 2008). The PM technology consists of several commercially available 96-well plates in which every well has a different substrate, allowing nearly 2,000 different microbial metabolic phenotypes to be tested (Bochner, 2003; Bochner, 2009; Bochner, Gadzinski & Panomitros, 2001; Bochner, Giovannetti & Viti, 2008). PM technology has been applied to several microorganisms, including mycobacteria (Baloni et al., 2014; Bochner, Giovannetti & Viti, 2008; Borglin et al., 2012; Chen, Scaria & Chang, 2012; Gupta, Kasetty & Chatterji, 2015; Johnson et al., 2008; Khatri et al., 2013; Lofthouse et al., 2013; Mackie et al., 2014; Mishra & Daniels, 2013; Nai et al., 2013; Omsland et al., 2009; Tohsato & Mori, 2008). One possible application of PM is the detection of phenotype changes due to gene knock-out. For example, Chen and co-authors showed that a *leuD* mutant of *M. avium* subsp *paratuberculosis* lost the ability to use several carbon, nitrogen, sulfur and phosphorous substrates (Chen, Scaria & Chang, 2012). Other researchers showed that the use of 12 carbon substrates differentiated *M. tuberculosis* from *M. bovis* (Khatri et al., 2013; Lofthouse et al., 2013).

In this study we tested clinical and environmental isolates of MAH using the PM technology. Our aim was to describe the metabolic substrates utilized by MAH isolates and to identify any metabolic differences between clinical and environmental MAH isolates.

## Materials and Methods

### Bacterial isolates and BIOLOG phenotype microarray

We analyzed five clinical and five environmental MAH isolates (Table 1).

**Table 1:** Characteristics of the ten MAH isolates analyzed in this study.

MAH Isolate name	Year of isolation	Source	Provider or reference	Accession of whole genome sequence
P-10091-06	2006	Clinical—Child with lymphadenitis	NRC for Mycobacteria, Borstel, Germany	LNAV00000000
2721	2004	Clinical—Child with lymphadenitis	NRC for Mycobacteria, Borstel, Germany	AWXJ00000000
P-9-13	2013	Clinical—Adult pulmonary infection	Charité Hospital, Berlin, Germany	LNBB00000000
104	1983	Clinical—Adult pulmonary infection	Reference strain, USA	CP000479
TH135	2013	Clinical—Adult pulmonary infection	Reference strain, Japan	AP012555
E-128	2010	Environmental—Soil	Friedrich Löffler Institute, Jena, Germany	LVCS00000000
E-96-2	2010	Environmental—Soil	This study	LMVW00000000
E- 82-7	2010	Environmental—Dust	This study	LNAF00000000
27-1	2010	Environmental—Dust	This study	AWXK00000000
E-2514	na	Environmental—Water	University of Düsseldorf, Germany	LNBJ00000000

**Notes.**

MAH *Mycobacterium avium* subsp. *hominissuis* NRC National reference center na Not available

We performed the BIOLOG Phenotype Microarray™ (BIOLOG, Hayward, CA, USA) according to the manufacturer’s recommendations. The technology is based on the measurement of bacterial respiration, which produces NADH (Bochner, Gadzinski & Panomitros, 2001). If bacteria are able to metabolize a specific substrate, electrons from NADH reduce a tetrazolium dye in an irreversible reaction generating a purple color in the PM plate wells. This color change is measured and recorded every 15 min by the reporter instrument OmniLog™ (BIOLOG, Hayward, CA, USA), generating a kinetic response curve for each well (Bochner, 2003; Bochner, 2009).

The ten MAH isolates were tested with the 96-wells plates PM1 to PM4, containing 190 carbon (PM1 and PM2), 95 nitrogen (PM3), 59 phosphorous (PM4) and 35 sulfur (PM4) substrates. The PM plates 1, 2 and 3 include one negative control well, in which bacteria are tested without any substrate. The PM4 plate includes two negative control wells, one for the phosphorus and one for the sulfur substrates. All isolates were tested three times. Briefly, we cultivated each MAH isolate in 30 ml of 7H10 Middlebrook medium supplemented with 10% modified ADC-enrichment (2% of glucose, 5% of BSA, 0,85% of NaCl) until an OD_600 nm_ of 0.3–0.6 was achieved (mid-logarithmic phase of growth). The use of liquid cultures in place of agar reduces bacterial clumping. Bacterial cultures were harvested by centrifugation for 10 min at 4,000 g and pellets were re-suspended in 10 ml of distilled water. Bacterial cells were starved for one night in water at room temperature to minimize false positive reactions due to nutrient accumulation in MAH cells and to ensure the use of the substrates provided by the PM plates. The following day the cells were centrifuged and re-suspended using a sterile stick in tubes containing 10 ml of GN/GP-IF-0a (BIOLOG inoculating fluid), 120 µl of 100× BIOLOG Redox Dye Mix G and 1 ml of the appropriate additive (Table 2), until 85% transmittance was reached as measured using the turbidimeter provided by BIOLOG. In order to reduce bacterial clumping, the sterile stick used for inoculation was ground against the wall of the tube. A volume of 100 *μ*l of this final suspension was added to each of the 96 wells of the PM plates. The PM plates were then sealed to avoid drying and incubated at 37 °C in the OmniLog^®^ (BIOLOG, Hayward, CA, USA) incubator reader for 8 days.

**Table 2:** Additives used for each PM plates. As additive are usually provided nutrient that are absent to the PM minimal media, but present in a standard MAH growth conditions. We used additives to make a complete minimal medium but omitted anything that could act as a source of the substrates of interest (for example, we did not include nitrate additives in the nitrogen source plates).

	Additive a	Additive b
PM plate usage	PM1, PM2, PM4	PM3
Ingredients	24 mM MgCl2	24 mM MgCl2
	12 mM CaCl2	12 mM CaCl2
	0,0012% ZnSO4	0,0012% ZnSO4
	0,06% ferric ammonium citrate	0,01% tween 80
	1,2% NH4Cl	
	0,01% tween 80	

As recommended by BIOLOG, we tested plates PM1 to PM4 using the same assay protocol but without the addition of bacteria in order to identify wells with abiotic dye reduction, which can generate false positive results.

### Analysis of BIOLOG phenotype microarray data

The raw kinetic data were exported as CSV files using OmniLog PM file Management/kinetic Analysis module (Bochner, 2003; Khatri et al., 2013). Differences in the metabolization of the different substrates by the ten MAH isolates were investigated by analyzing the maximum height of the bacterial respiration curves (parameter A) using the R-package opm (Vaas et al., 2013). To allow comparisons across plates processed in different experimental runs, the A parameters were normalized by subtracting the well mean of the negative control (Vaas et al., 2013). Furthermore, the A parameters of the triplicates were combined by calculating the mean and discretized into “positive,” “moderate” and “negative” metabolization using the method “discrete” within the R-opm package. Substrates differentiating the isolates from each other were visualized as a heatmap generated using the R-packages heatmap.plus with the Euclidean algorithm. The heatmap displays the utilization of each substrate with a color key: yellow for strong positive metabolization, green for moderate metabolization and blue for no metabolization.

### Analysis of metabolic pathways

The metabolic pathways of the two substrates of interest butyric and propionic acid have been further analyzed. Specifically, we extracted all sequences of the genes known to be associated with the pathways related to butyric and propionic acid from the KEGG pathway database (Kanehisa et al., 2016). We extracted the genes from all the *M. avium* subspecies (*n* = 8) present in the KEGG pathway database, namely: *M. avium* subsp. *paratuberculosis* K-10, *M. avium* subsp. *paratuberculosis* MAP4, *M. avium* subsp. *paratuberculosis* E1, *M. avium* subsp. *paratuberculosis* E93, *M. avium* subsp. *avium* DJO-44271, *M. avium* subsp. *avium* 2285 (R), *M. avium* subsp. *avium* 2285 (S) and the *M. avium* 104. The redundant genes have been excluded. Then we screened all such genes in genomes of our ten MAH isolates by performing a Custom BLAST analysis using Geneious version 9 (Kearse et al., 2012). The parameters for the screening that we used to determine if a gene was present or not were: sequence identity ≥90%, sequence coverage ≥90%, *e* value ≤0.01.

In addition, we analyzed the number of Single Nucleotide polymorphisms (SNP)s (both synonymous and nonsynonymous) in the sequence of the genes detected in our MAH isolates. For each gene, we also constructed a phylogenetic tree using the nucleotide sequences to determine whether any SNP was associated with clinical or environmental source of the isolates based on the Tamura–Nei model using Geneious version 9.

### Statistical analyses

We generated two groups, one with data from all clinical isolates and the other with data from all environmental isolates. Statistical differences between clinical and environmental isolates in the metabolization of butyric acid and propionic acid were evaluated by means of 95% family-wise comparison of group means (Tukey contrast test) of the parameter A on specific wells using the function “opm_mcp” within the opm R-package. A *p* value less than 0.05 was considered to be statistically significant.

### Whole genome sequencing of MAH isolates

Genomic DNAs were extracted from the MAH isolates as described previously (Lewin et al., 2003). Whole genome sequencing (WGS) was performed using Illumina MiSeq 300 bp paired-end sequencing, yielding a coverage that exceeded 100×. The NGS QC tool kit was used to assess the quality of the data reads, which was set as reads with a minimum of 70% of bases having a phred score greater than 20 (Patel & Jain, 2012). De novo assembly of the resulting reads into multiple contigs was performed using CLC Genomics Workbench 8.0 (CLC bio, Aarhus, Denmark) and contigs annotation was done using RAST (Aziz et al., 2008).

### Determination of the maximum common genome and of the accessory genome

We determined the maximum common genome (MCG), comprising those genes present in all of the ten MAH genomes, as reported previously (Von Mentzer et al., 2014). All these genes were then extracted from all genomes, concatenated and aligned. The resulting alignment was used to generate a clustering tree using RAxML 8.1 (Stamatakis, 2014).

For determination of the accessory genome we applied the PanGenome Pipeline –Roary. After determination of the accessory genome of the ten MAH genomes and its distribution within them, we separated those genes that are exclusively present only in either the environmental strains or the clinical strains (Page et al., 2015).

## Results

### Substrate utilization of the ten MAH isolates

We tested the capability of our ten MAH isolates to metabolize 379 different substrates. In total, 334/379 (88.1%) substrates were negative for all of the isolates (see Table S1). A total of 23/379 (6.1%) substrates caused abiotic reactions and were excluded from further analysis. A list of false-positive substrates is shown in the Table S2. The kinetic curves corresponding to the control plates PM1 to PM4 tested without bacteria are presented in the Fig. S1.

**Figure 1:**
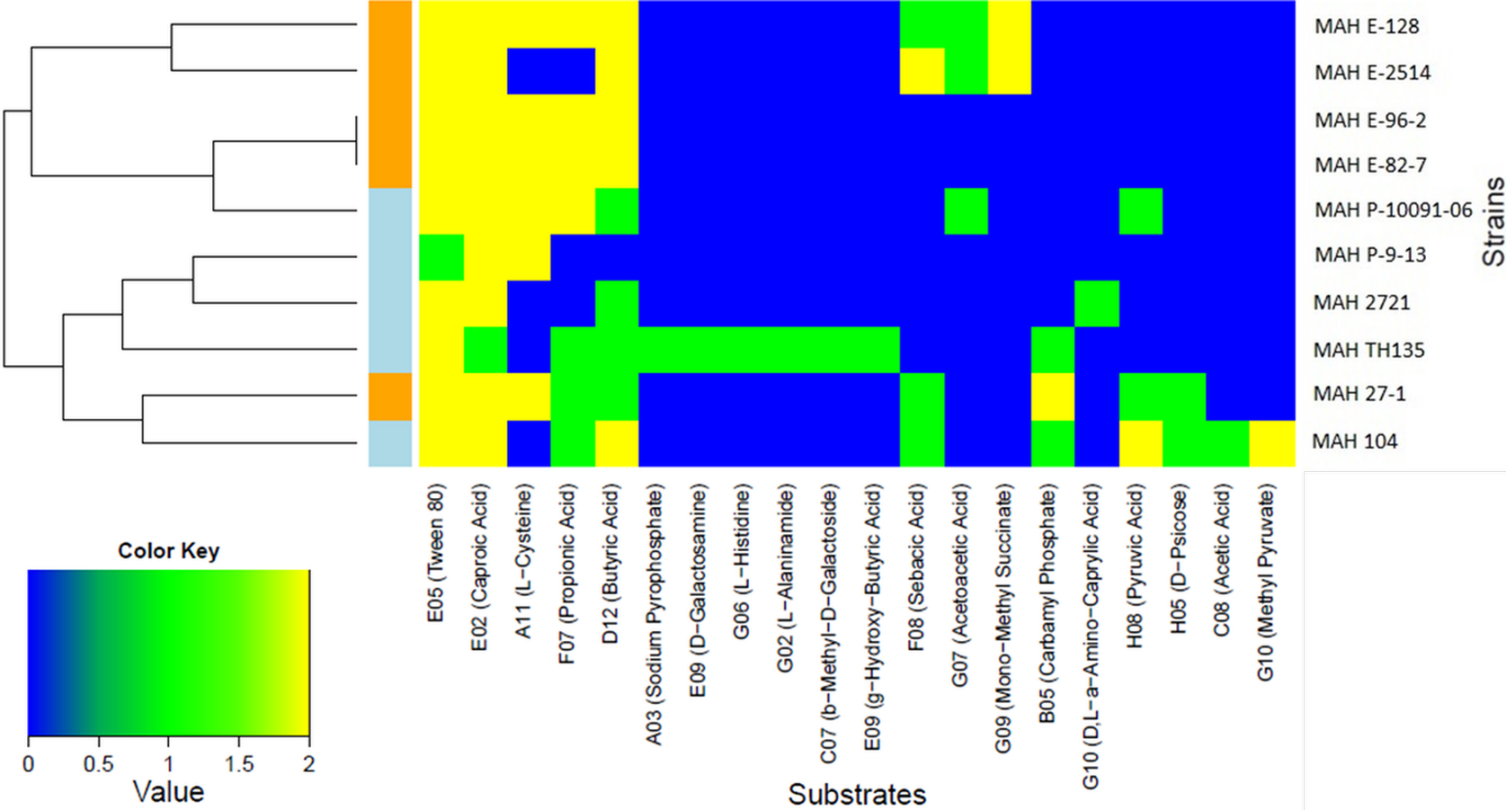
Heatmap showing the 20 substrates that were differently metabolized by the ten MAH isolates analyzed in this study. The color key scale for each substrate is based on dye reduction quantified by Omnilog units. A yellow color indicates strong positive substrate metabolization, a green color moderate metabolization and a blue color indicates no substrate metabolization. Regarding the MAH isolates, environmental isolates are marked in orange, while clinical isolates are marked in blue.

Only two carbon substrates, the fatty acid derivatives Tween 20 and Tween 40 were strongly positive for all of the ten MAH isolates. The kinetic curves for these substrates reached 250 Omnilog units, amongst the highest values recorded in our analysis (see Fig. S2 for all kinetic curves of the ten MAH isolates). The opm analysis revealed that a total of 20/379 (5.3%) substrates were metabolized differently among the MAH isolates (Table 3). We therefore carried out further analysis using only these substrates. The majority of these 20 substrates were carbon substrates, 15/20 (75.0%), followed by 3 nitrogen and 2 phosphorous substrates. The heatmap in Fig. 1 shows the utilization of these 20 substrates among the ten MAH isolates. The isolates are grouped according to their substrate utilization. Isolates utilizing similar substrates appear to cluster together.

**Table 3:** The 20 substrates differentiating the ten MAH isolates analyzed in this study.

PM Plate	Substrate and well number	Pathway involved	Reference
PM1 Carbon	Acetic acid –C08	Pyruvate metabolism	Baloni et al. (2014), Nai et al. (2013)
	Acetoacetic acid –G07	Pyruvate metabolism	Baloni et al. (2014), Nai et al. (2013)
	Methyl pyruvate –G10	Pyruvate metabolism	Baloni et al. (2014), Nai et al. (2013)
	Mono–methyl Succinate –G09	Tricarboxylic acid cycle	Baloni et al. (2014), Nai et al. (2013)
	Propionic acid –F07	Propanoate metabolism, Nicotinate and nicotinamide metabolism, Degradation of aromatic compounds	Baloni et al. (2014), Kanehisa & Goto (2000), Kanehisa et al. (2016), Nai et al. (2013)
	D-psicose –H05	Glycolysis and branches	Baloni et al. (2014), Nai et al. (2013)
	Pyruvic acid –H08	Pyruvate metabolism	Baloni et al. (2014), Nai et al. (2013)
	Tween 80 –E05	Fatty acid metabolism	Baloni et al. (2014), Nai et al. (2013)
PM2 Carbon	L-alaninamide –G02	Amino acid metabolism	Nai et al. (2013)
	Butyric acid –D12	Butanoate metabolism	Baloni et al. (2014), Kanehisa & Goto (2000), Kanehisa et al. (2016), Nai et al. (2013)
	Caproic acid –E02	Carboxylic acid metabolism	Nai et al. (2013)
	L-histidine –G06	Amino acid metabolism	Nai et al. (2013)
	*γ*-hydroxy-butyric acid –E09	Succinate metabolism	Breitkreuz et al. (2003), Nai et al. (2013)
	*β* -methyl-D-galactoside –C07	Galactose Metabolism	Nai et al. (2013)
	Sebacic acid –F08	Carboxylic acid metabolism	Nai et al. (2013)
PM3 Nitrogen	D,L-*α* -amino-caprylic acid –G10	Amino acid metabolism	Baloni et al. (2014)
	L-cysteine –A11	Amino acid metabolism	Baloni et al. (2014)
	D-galactosamine –E09	Amino-sugar pathway	Baloni et al. (2014)
PM4 Phosphorous and sulphur	Carbamyl phosphate –B05	Urea cycle and Pyrimidine synthesis	Nelson & Cox (2004)
	Sodium pyrophosphate –A03	Phosphoric acid synthesis	Nelson & Cox (2004)

Two major clusters, each composed of five isolates, could be observed. One was rich in environmental isolates (4/5) and the other was rich in clinical isolates (4/5). The substrates predominantly contributing to this clustering were butyric acid and propionic acid and indeed, the Tukey’s test revealed that environmental isolates metabolized more strongly butyric acid (*p* = 0.0209) and propionic acid (*p* = 0.00307) than clinical isolates with statistical significance.

### Metabolic pathways analysis

The propionic and butyric acid are involved in three and one pathway, respectively (Table 3). A total of 151 genes have been identified in the KEGG database associated with all these pathways (Kanehisa et al., 2016). In Table S3 we reported the distribution and SNPs analysis of those genes in the MAH genomes. Of the 151 genes, 134 (88.7%) are present in all the ten MAH. The median gene length was 1,099 bp (range 318–2,253), whereas the median number of SNPs per gene is 17 (range 1–147). The phylogenetic analysis revealed that none of the SNPs could be associated with the group of the clinical or the group of environmental MAH isolates (see Table S4). In the propanoate pathway four operons have been identified: *fadAB* associated with the *β*-oxidation of several fatty acids (DiRusso, 1990), *ech8*-*9* encoding for hydrogenases that play a role in energy conversion (Sant’Anna et al., 2015), *sucCD* responsible for the succinate metabolism (Cerdeno-Tarraga et al., 2003) and *mutAB* involved in the methylmalonate pathway (Schoenwolf & Alvarez, 1989). In the nicotinate pathway there are two operons: the *pntAA-AB-B* responsible for the transhydrogenation between NADH and NADP (Anderlund et al., 1999) and *nadABC* involved in the biosynthesis of NAD+ (Vilcheze et al., 2010). In the degradation of aromatic compounds pathway we identified the *pcaHGB* operon involved in the *β*-ketoadipate pathway (Harwood & Parales, 1996). In the butanoate pathway we identified the *fadAB* and *ech8*-*9operons*, the *sdhCDAB* encoding for the succinate dehydrogenase complex involved in the fatty acid metabolism (Nam et al., 2005) and the *ilvBN* responsible for the acetolactate synthesis, a precursor of several amino acids (Keilhauer, Eggeling & Sahm, 1993).

### Clustering analysis and determination of the accessory genome

**Figure 2:**
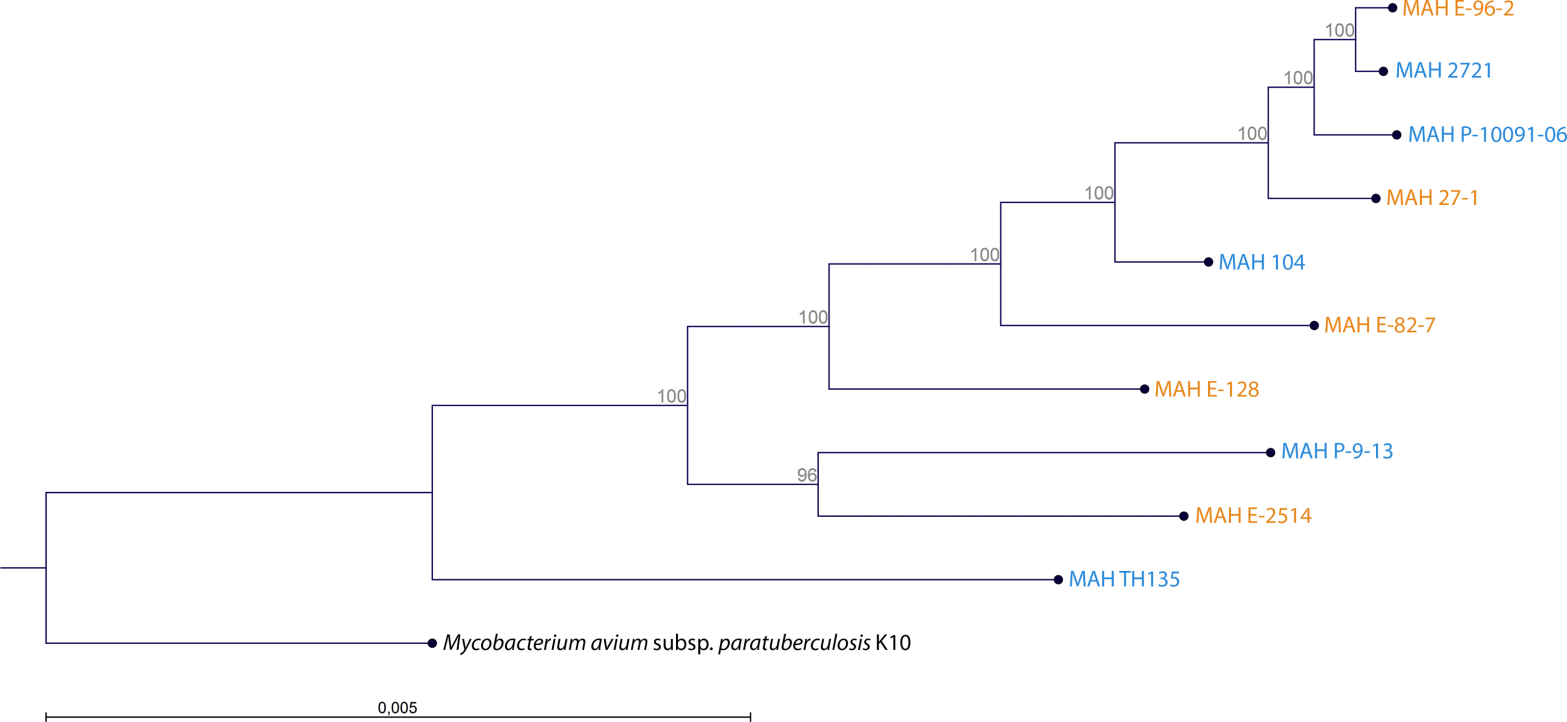
Clustering of the 10 MAH isolates. The tree was generated using RAxML 8.1. The alignment comprised 1,658 genes constituting the maximum common genome of our ten MAH isolates. Two reference strains were also included (MAH 104 and MAH TH135). The genome sequence of *M. avium* subsp. *paratuberculosis* K10 (Accession Number: AE016958) was used as outgroup. Isolate origin is also represented by blue for clinical origin and orange for environmental origin. The percentage of trees in which the associated taxa clustered together is shown adjacent to the branches.

The WGS of the two reference strains MAH 104 and MAH TH135 were already in the GenBank database and we submitted the remaining genomes at DDBJ/EMBL/GenBank under the BioProject Number PRJNA299461. The MCG, the maximum number of genes shared by all ten MAH isolates was 1,658, the alignment of which spanned 1.378 Mbp. The clustering analysis of the ten MAH isolates is shown in Fig. 2. By comparing the genetic clustering obtained by WGS with the phenotypic clustering obtained through BIOLOG PM we observed slight differences. For examples, the isolates MAH E-96-2 and MAH E-82-7, which share identical metabolic profiles, were genetically more distant from each other. Interestingly, at the genetic level there was no obvious clustering between the group of clinical and the group of environmental isolates.

The accessory genome is constituted by 4,067 genes. A total of 1,688 genes were specific for the group of clinical isolates (Table S5). On the other hand, 698 genes were specific for the group of environmental isolates (Table S6). We found no genes that were present in all the clinical and absent in all the environmental isolates, and vice-versa. Among the most abundant specific genes of the two groups of isolates, there were no known genes associated with the pathways which involved butyric and propionic acid. However, genes annotated as hypothetical proteins represented the most abundant specific genes of the two groups

## Discussion

This study represents the first phenotypic analysis of a collection of clinical and environmental MAH isolates using the Biolog PM technology. We showed that the PM technology works well and can be performed with MAH isolates. Strong positive reactions with several substrates were observed with kinetic curves exceeding 200 Omnilog dye units. Although some substrates were metabolized only moderately by our MAH isolates (green in Fig. 1), this might be due to the fact that the use of such substrates by bacteria has a time lag.

The ten MAH isolates showed different metabolic patterns pointing to high intra-species diversity. Only two out of the ten isolates had identical heatmap profiles (MAH E-96-2 and MAH E-82-7).

Our study showed that MAH isolates prefer to metabolize fatty acids as a carbon source. Indeed, the Tween substrates were strongly metabolized by all MAH isolates tested. This is in agreement with prior studies, showing that Tween substrates were widely used by different mycobacterial species (Baloni et al., 2014; Chen, Scaria & Chang, 2012; Hayashi et al., 2010; Khatri et al., 2013; Lofthouse et al., 2013; Wang et al., 2011). It has been reported that mycobacteria hydrolyze Tween 80 to generate the fatty acid oleic acid, which can enter the Tricarboxylic acid (TCA) cycle or can be used as a substrate for energy production (Lofthouse et al., 2013; Vandal, Nathan & Ehrt, 2009). Other fatty acids used by the majority of our MAH isolates are represented by two short fatty acids, caproic acid and butyric acid (Kanehisa & Goto, 2000; Kanehisa et al., 2016; Khatri et al., 2013). Caproic acid and its derivatives are involved in several mycobacterial pathways such as the degradation of aromatic compounds, oxocarboxylic acid metabolism or lysine degradation (Kanehisa & Goto, 2000; Kanehisa et al., 2016). The butyric acid is the final product of butanoate metabolism. Propionic acid is another fatty acid used by our MAH isolates and this represents the terminal product of propanoate metabolism (Kanehisa & Goto, 2000; Kanehisa et al., 2016). The nitrogen source L-cysteine, used by six of our MAH isolates, is the final product of cysteine metabolism and is involved in the biosynthesis of other amino acids such as methionine and histidine (Baloni et al., 2014; Kanehisa & Goto, 2000; Kanehisa et al., 2016).

The question of whether bacteria of the same species originating from either clinical or environmental sources differ from each other is still a matter of discussion. Li and co-authors (2014) showed that comparative genome analysis clearly distinguished clinical and environmental *Vibrio parahaemolyticus* isolates from each other. In contrast, other researchers have reported no difference between clinical and environmental *Pseudomonas aeruginosa* isolates with regard to virulence and metabolic properties (Alonso, Rojo & Martinez, 1999; Vives-Florez & Garnica, 2006). Although our study did not reveal any clear distinction between clinical or environmental MAH isolates at the level of the whole genome, we observed differences between clinical and environmental isolates with regard to substrate utilization. The most intriguing difference is that the two fatty acids butyric acid and propionic acid are metabolized more by the environmental than by clinical isolates.

We observed no difference in the presence / absence of genes associated with butyric or propionic acid pathways among the group of clinical and the group of environmental MAH isolates. The SNPs analysis of the genes involved in the pathways revealed that no SNPs were associated with clinical or environmental origin of the MAH isolates. These evidences suggest that the metabolic differences observed among clinical and environmental MAH isolates might be due to difference in gene regulation. However, we screened all the genes that up to now have been associated with the pathways of interest. We can speculate that there might be additional genes, of unknown function, that might play a role in the above pathways.

The analysis of the accessory genome revealed that none of the genes specific for the clinical or for the environmental isolates could be associated with the pathways of interest. However, future studies on the high number of hypothetical proteins might clarify whether they have a role in the pathways which involved the butyric and propionic acid.

The higher metabolic activity observed among environmental MAH isolates might be advantageous for survival in an environment presenting a wider range of nutritional conditions than the host cells alone. Further studies testing a larger number of isolates from different origins might clarify this. In addition, it has been showed that in bacteria the fatty acids have a role in adaptation to different environmental conditions (De Sarrau et al., 2012; De Sarrau et al., 2013; Diomande et al., 2015).

## Conclusions

Our study contributes to the understanding of the emerging pathogen MAH at the phenotypic and metabolic level. Understanding how bacteria utilize their own or host-derived substrates during infection might help the development of strategies to fight such infections. We encourage phenotypic testing of microbial isolates from different ecological niches to identify key substrates or pathways that can be used as targets for drug development or for selective growth media development.

##  Supplemental Information

10.7717/peerj.2833/supp-1 Figure S1 Kinetic curves corresponding to the plates PM1 to PM4 tested without any bacteria In the X-axis time is reported. In the Y-axis Omnilog units are reported. The Omnilog unit is a measurement of dye reduction and therefore a measurement of bacterial respiration. Wells in which the dye reduction is observed usually became positive soon and have a flat line over the time.

10.7717/peerj.2833/supp-2 Figure S2 Kinetic curves corresponding to the plates PM1 to PM4 tested with the ten MAH isolates analyzed in this study In the X-axis time is reported. In the Y-axis Omnilog units are reported. The Omnilog unit is a measurement of dye reduction and therefore a measurement of bacterial respiration. Each color represents a different isolate. For each isolate, the median value among the three replicates is given.

10.7717/peerj.2833/supp-3 Table S1 The 334 substrates showing negative reaction for all of the ten MAH isolates tested, listed by PM plates

10.7717/peerj.2833/supp-4 Table S2 List of the 23 wells causing abiotic dye reduction The number and letter of each substrate indicate the exact position in the PM plate.

10.7717/peerj.2833/supp-5 Table S3 Distribution of the genes associated with the propionic acid and the butyric acid pathways among the ten MAH isolates The three pathways where the propionic acid is involved, and the one pathway where the butirric acid is invovled are shown.

10.7717/peerj.2833/supp-6 Table S4 Phylogenetic analysis of each gene associated with the propionic acid and the butyric acid pathways among the ten MAH isolates For each gene we made a phylogenetic tree based on the nucleotide sequences. The gene analyzed are the ones presented in the Table S3. in blue clinical isolates, in orange environmental isolates are shown

10.7717/peerj.2833/supp-7 Table S5 Distribution of the 1,688 genes specific for the clinical isolates of *Mycobacterium avium* subsp. *hominissuis(MAH)* The presence or abscence of the 1,688 genes in the five clinical MAH isolates is shown. The genes are ordered by frequency. The number of genes specific and unique for each clinical isolate is provided.

10.7717/peerj.2833/supp-8 Table S6 Distribution of the 698 genes specific for the environmental isolates of *Mycobacterium avium* subsp. *hominissuis(MAH)* The presence or abscence of the 698 genes in the five clinical MAH isolates is shown. The genes are ordered by frequency. The number of genes specific and unique for each environmental isolate is provided.

## Additional Information and Declarations

### Data Availability

The following information was supplied regarding data availability: The raw data has been supplied as Supplementary Files.

## References

[ref-1] Alonso A, Rojo F, Martinez JL (1999). Environmental and clinical isolates of *Pseudomonas aeruginosa* show pathogenic and biodegradative properties irrespective of their origin. Environmental Microbiology.

[ref-2] Anderlund M, Nissen TL, Nielsen J, Villadsen J, Rydstrom J, Hahn-Hagerdal B, Kielland-Brandt MC (1999). Expression of the *Escherichia coli* pntA and pntB genes, encoding nicotinamide nucleotide transhydrogenase, in Saccharomyces cerevisiae and its effect on product formation during anaerobic glucose fermentation. Applied and Environmental Microbiology.

[ref-3] Aziz RK, Bartels D, Best AA, DeJongh M, Disz T, Edwards RA, Formsma K, Gerdes S, Glass EM, Kubal M, Meyer F, Olsen GJ, Olson R, Osterman AL, Overbeek RA, McNeil LK, Paarmann D, Paczian T, Parrello B, Pusch GD, Reich C, Stevens R, Vassieva O, Vonstein V, Wilke A, Zagnitko O (2008). The RAST server: rapid annotations using subsystems technology. BMC Genomics.

[ref-4] Baloni P, Padiadpu J, Singh A, Gupta KR, Chandra N (2014). Identifying feasible metabolic routes in *Mycobacterium smegmatis* and possible alterations under diverse nutrient conditions. BMC Microbiology.

[ref-5] Bannantine JP, Bayles DO, Robbe-Austerman S, Burrell AM, Stabel JR (2014). Draft genome sequence of a *Mycobacterium avium* complex isolate from a Broadbill Bird. Genome Announc.

[ref-6] Bochner BR (2003). New technologies to assess genotype-phenotype relationships. Nature Reviews Genetics.

[ref-7] Bochner BR (2009). Global phenotypic characterization of bacteria. FEMS Microbiology Reviews.

[ref-8] Bochner BR, Gadzinski P, Panomitros E (2001). Phenotype microarrays for high-throughput phenotypic testing and assay of gene function. Genome Research.

[ref-9] Bochner BR, Giovannetti L, Viti C (2008). Important discoveries from analysing bacterial phenotypes. Molecular Microbiology.

[ref-10] Borglin S, Joyner D, DeAngelis KM, Khudyakov J, D’Haeseleer P, Joachimiak MP, Hazen T (2012). Application of phenotypic microarrays to environmental microbiology. Current Opinion in Biotechnology.

[ref-56] Breitkreuz KE, Allan WL, Van Cauwenberghe OR, Jakobs C, Talibi D, Andre B, Shelp BJ (2003). A novel gamma-hydroxybutyrate dehydrogenase: identification and expression of an Arabidopsis cDNA and potential role under oxygen deficiency. Journal of Biological Chemistry.

[ref-11] Cerdeno-Tarraga AM, Efstratiou A, Dover LG, Holden MT, Pallen M, Bentley SD, Besra GS, Churcher C, James KD, De Zoysa A, Chillingworth T, Cronin A, Dowd L, Feltwell T, Hamlin N, Holroyd S, Jagels K, Moule S, Quail MA, Rabbinowitsch E, Rutherford KM, Thomson NR, Unwin L, Whitehead S, Barrell BG, Parkhill J (2003). The complete genome sequence and analysis of Corynebacterium diphtheriae NCTC13129. Nucleic Acids Research.

[ref-12] Chen JW, Scaria J, Chang YF (2012). Phenotypic and transcriptomic response of auxotrophic *Mycobacterium avium* subsp. *paratuberculosis* leuD mutant under environmental stress. PLoS ONE.

[ref-13] De Sarrau B, Clavel T, Clerte C, Carlin F, Ginies C, Nguyen-The C (2012). Influence of anaerobiosis and low temperature on Bacillus cereus growth, metabolism, and membrane properties. Applied and Environmental Microbiology.

[ref-14] De Sarrau B, Clavel T, Zwickel N, Despres J, Dupont S, Beney L, Tourdot-Marechal R, Nguyen-The C (2013). Unsaturated fatty acids from food and in the growth medium improve growth of Bacillus cereus under cold and anaerobic conditions. Food Microbiology.

[ref-15] Despierres L, Cohen-Bacrie S, Richet H, Drancourt M (2012). Diversity of *Mycobacterium avium* subsp. *hominissuis* mycobacteria causing lymphadenitis, France. European Journal of Clinical Microbiology and Infectious Diseases.

[ref-16] Diomande SE, Nguyen-The C, Guinebretiere MH, Broussolle V, Brillard J (2015). Role of fatty acids in Bacillus environmental adaptation. Frontiers in Microbiology.

[ref-17] DiRusso CC (1990). Primary sequence of the *Escherichia coli* fadBA operon, encoding the fatty acid-oxidizing multienzyme complex, indicates a high degree of homology to eucaryotic enzymes. Journal of Bacteriology.

[ref-18] Falkinham 3rd JO (2013). Ecology of nontuberculous mycobacteria–where do human infections come from?. Semin Respir Crit Care Med.

[ref-19] Gupta KR, Kasetty S, Chatterji D (2015). Novel functions of (p)ppGpp and Cyclic di-GMP in mycobacterial physiology revealed by phenotype microarray analysis of wild-type and isogenic strains of *Mycobacterium smegmatis*. Applied and Environmental Microbiology.

[ref-20] Harwood CS, Parales RE (1996). The beta-ketoadipate pathway and the biology of self-identity. Annual Review of Microbiology.

[ref-21] Hayashi D, Takii T, Mukai T, Makino M, Yasuda E, Horita Y, Yamamoto R, Fujiwara A, Kanai K, Kondo M, Kawarazaki A, Yano I, Yamamoto S, Onozaki K (2010). Biochemical characteristics among *Mycobacterium bovis* BCG substrains. FEMS Microbiology Letters.

[ref-22] Hoefsloot W, Van Ingen J, Andrejak C, Angeby K, Bauriaud R, Bemer P, Beylis N, Boeree MJ, Cacho J, Chihota V, Chimara E, Churchyard G, Cias R, Daza R, Daley CL, Dekhuijzen PN, Domingo D, Drobniewski F, Esteban J, Fauville-Dufaux M, Folkvardsen DB, Gibbons N, Gomez-Mampaso E, Gonzalez R, Hoffmann H, Hsueh PR, Indra A, Jagielski T, Jamieson F, Jankovic M, Jong E, Keane J, Koh WJ, Lange B, Leao S, Macedo R, Mannsaker T, Marras TK, Maugein J, Milburn HJ, Mlinko T, Morcillo N, Morimoto K, Papaventsis D, Palenque E, Paez-Pena M, Piersimoni C, Polanova M, Rastogi N, Richter E, Ruiz-Serrano MJ, Silva A, Da Silva MP, Simsek H, Van Soolingen D, Szabo N, Thomson R, Tortola Fernandez T, Tortoli E, Totten SE, Tyrrell G, Vasankari T, Villar M, Walkiewicz R, Winthrop KL, Wagner D, Nontuberculous Mycobacteria Network European Trials G (2013). The geographic diversity of nontuberculous mycobacteria isolated from pulmonary samples: an NTM-NET collaborative study. European Respiratory Journal.

[ref-23] Johnson DA, Tetu SG, Phillippy K, Chen J, Ren Q, Paulsen IT (2008). High-throughput phenotypic characterization of Pseudomonas aeruginosa membrane transport genes. PLoS Genet.

[ref-24] Kanehisa M, Goto S (2000). KEGG: kyoto encyclopedia of genes and genomes. Nucleic Acids Research.

[ref-25] Kanehisa M, Sato Y, Kawashima M, Furumichi M, Tanabe M (2016). KEGG as a reference resource for gene and protein annotation. Nucleic Acids Research.

[ref-26] Kearse M, Moir R, Wilson A, Stones-Havas S, Cheung M, Sturrock S, Buxton S, Cooper A, Markowitz S, Duran C, Thierer T, Ashton B, Meintjes P, Drummond A (2012). Geneious basic: an integrated and extendable desktop software platform for the organization and analysis of sequence data. Bioinformatics.

[ref-27] Keilhauer C, Eggeling L, Sahm H (1993). Isoleucine synthesis in Corynebacterium glutamicum: molecular analysis of the ilvB-ilvN-ilvC operon. Journal of Bacteriology.

[ref-28] Khatri B, Fielder M, Jones G, Newell W, Abu-Oun M, Wheeler PR (2013). High throughput phenotypic analysis of *Mycobacterium tuberculosis* and *Mycobacterium bovis* strains’ metabolism using biolog phenotype microarrays. PLoS ONE.

[ref-29] Kim BJ, Choi BS, Lim JS, Choi IY, Lee JH, Chun J, Kook YH, Kim BJ (2012). Complete genome sequence of Mycobacterium intracellulare strain ATCC 13950(T). Journal of Bacteriology.

[ref-30] Lahiri A, Kneisel J, Kloster I, Kamal E, Lewin A (2014). Abundance of *Mycobacterium avium* ssp. *hominissuis* in soil and dust in Germany—implications for the infection route. Letters in Applied Microbiology.

[ref-31] Lewin A, Freytag B, Meister B, Sharbati-Tehrani S, Schafer H, Appel B (2003). Use of a quantitative TaqMan-PCR for the fast quantification of mycobacteria in broth culture, eukaryotic cell culture and tissue. J Vet Med B Infect Dis Vet Public Health.

[ref-32] Li L, Wong HC, Nong W, Cheung MK, Law PT, Kam KM, Kwan HS (2014). Comparative genomic analysis of clinical and environmental strains provides insight into the pathogenicity and evolution of Vibrio parahaemolyticus. BMC Genomics.

[ref-33] Lofthouse EK, Wheeler PR, Beste DJ, Khatri BL, Wu H, Mendum TA, Kierzek AM, McFadden J (2013). Systems-based approaches to probing metabolic variation within the *Mycobacterium tuberculosis* complex. PLoS ONE.

[ref-34] Mackie AM, Hassan KA, Paulsen IT, Tetu SG (2014). Biolog Phenotype Microarrays for phenotypic characterization of microbial cells. Methods in Molecular Biology.

[ref-36] Mishra MN, Daniels L (2013). Characterization of the MSMEG_2631 gene (mmp) encoding a multidrug and toxic compound extrusion (MATE) family protein in *Mycobacterium smegmatis* and exploration of its polyspecific nature using biolog phenotype microarray. Journal of Bacteriology.

[ref-37] Nai C, Wong HY, Pannenbecker A, Broughton WJ, Benoit I, De Vries RP, Gueidan C, Gorbushina AA (2013). Nutritional physiology of a rock-inhabiting, model microcolonial fungus from an ancestral lineage of the Chaetothyriales (Ascomycetes). Fungal Genetics and Biology.

[ref-38] Nam TW, Park YH, Jeong HJ, Ryu S, Seok YJ (2005). Glucose repression of the *Escherichia coli* sdhCDAB operon, revisited: regulation by the CRP*cAMP complex. Nucleic Acids Research.

[ref-57] Nelson DL, Cox MM (2004). Lehninger principles of biochemistry.

[ref-39] Omsland A, Cockrell DC, Howe D, Fischer ER, Virtaneva K, Sturdevant DE, Porcella SF, Heinzen RA (2009). Host cell-free growth of the Q fever bacterium Coxiella burnetii. Proceedings of the National Academy of Sciences of the United States of America.

[ref-40] Page AJ, Cummins CA, Hunt M, Wong VK, Reuter S, Holden MT, Fookes M, Falush D, Keane JA, Parkhill J (2015). Roary: rapid large-scale prokaryote pan genome analysis. Bioinformatics.

[ref-41] Patel RK, Jain M (2012). NGS QC Toolkit: a toolkit for quality control of next generation sequencing data. PLoS ONE.

[ref-42] Plata G, Henry CS, Vitkup D (2015). Long-term phenotypic evolution of bacteria. Nature.

[ref-43] Rindi L, Garzelli C (2014). Genetic diversity and phylogeny of *Mycobacterium avium*. Infection, Genetics and Evolution.

[ref-44] Sant’Anna FH, Lebedinsky AV, Sokolova TG, Robb FT, Gonzalez JM (2015). Analysis of three genomes within the thermophilic bacterial species Caldanaerobacter subterraneus with a focus on carbon monoxide dehydrogenase evolution and hydrolase diversity. BMC Genomics.

[ref-45] Schoenwolf GC, Alvarez IS (1989). Roles of neuroepithelial cell rearrangement and division in shaping of the avian neural plate. Development.

[ref-46] Stamatakis A (2014). RAxML version 8: a tool for phylogenetic analysis and post-analysis of large phylogenies. Bioinformatics.

[ref-47] Tohsato Y, Mori H (2008). Phenotype profiling of single gene deletion mutants of *E. coli* using Biolog technology. Genome Inform.

[ref-48] Tortoli E (2014). Microbiological features and clinical relevance of new species of the genus *Mycobacterium*. Clinical Microbiology Reviews.

[ref-49] Uchiya K, Takahashi H, Yagi T, Moriyama M, Inagaki T, Ichikawa K, Nakagawa T, Nikai T, Ogawa K (2013). Comparative genome analysis of *Mycobacterium avium* revealed genetic diversity in strains that cause pulmonary and disseminated disease. PLoS ONE.

[ref-50] Vaas LA, Sikorski J, Hofner B, Fiebig A, Buddruhs N, Klenk HP, Goker M (2013). opm: an R package for analysing OmniLog(R) phenotype microarray data. Bioinformatics.

[ref-51] Vandal OH, Nathan CF, Ehrt S (2009). Acid resistance in *Mycobacterium tuberculosis*. Journal of Bacteriology.

[ref-52] Vilcheze C, Weinrick B, Wong KW, Chen B, Jacobs Jr WR (2010). NAD+ auxotrophy is bacteriocidal for the tubercle bacilli. Molecular Microbiology.

[ref-53] Vives-Florez M, Garnica D (2006). Comparison of virulence between clinical and environmental Pseudomonas aeruginosa isolates. Int Microbiol.

[ref-35] Von Mentzer A, Connor TR, Wieler LH, Semmler T, Iguchi A, Thomson NR, Rasko DA, Joffre E, Corander J, Pickard D, Wiklund G, Svennerholm AM, Sjoling A, Dougan G (2014). Identification of enterotoxigenic *Escherichia coli* (ETEC) clades with long-term global distribution. Nature Genetics.

[ref-54] Wang C, Mahrous EA, Lee RE, Vestling MM, Takayama K (2011). Novel polyoxyethylene-containing glycolipids are synthesized in Corynebacterium matruchotii and *Mycobacterium smegmatis* cultured in the presence of tween 80. J Lipids.

[ref-55] Wynne JW, Seemann T, Bulach DM, Coutts SA, Talaat AM, Michalski WP (2010). Resequencing the *Mycobacterium avium* subsp. *paratuberculosis* K10 genome: improved annotation and revised genome sequence. Journal of Bacteriology.

