# Supplementary figures and images for: Metabolic phenotype of clinical and environmental Mycobacterium avium subsp. hominissuis isolates
Source: PeerJ. 2017 Jan 3;5:e2833. doi: 10.7717/peerj.2833 (PMC5214758; doi:10.7717/peerj.2833)

# PM01 (Carbon Sources)

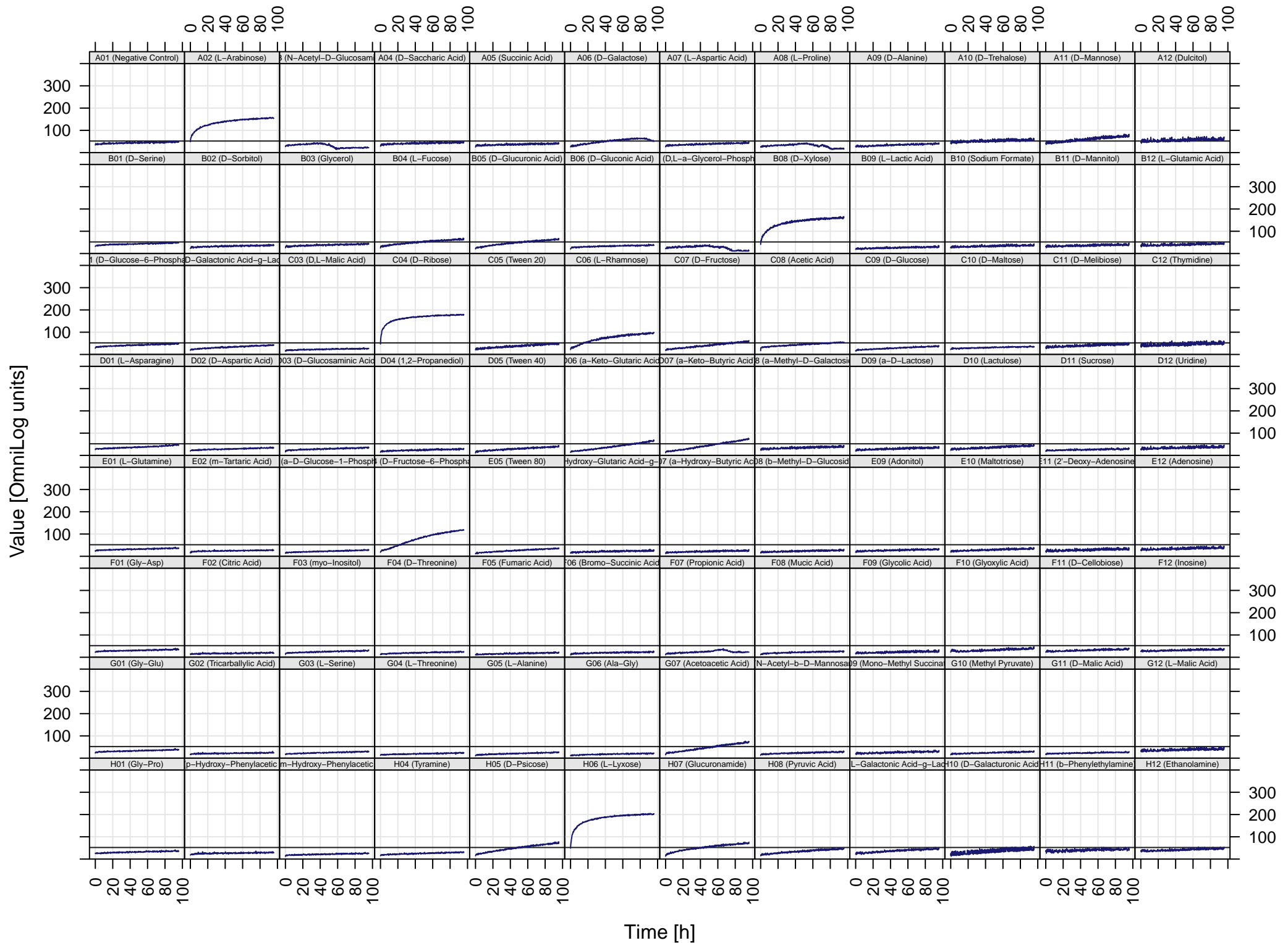

# PM02 (Carbon Sources)

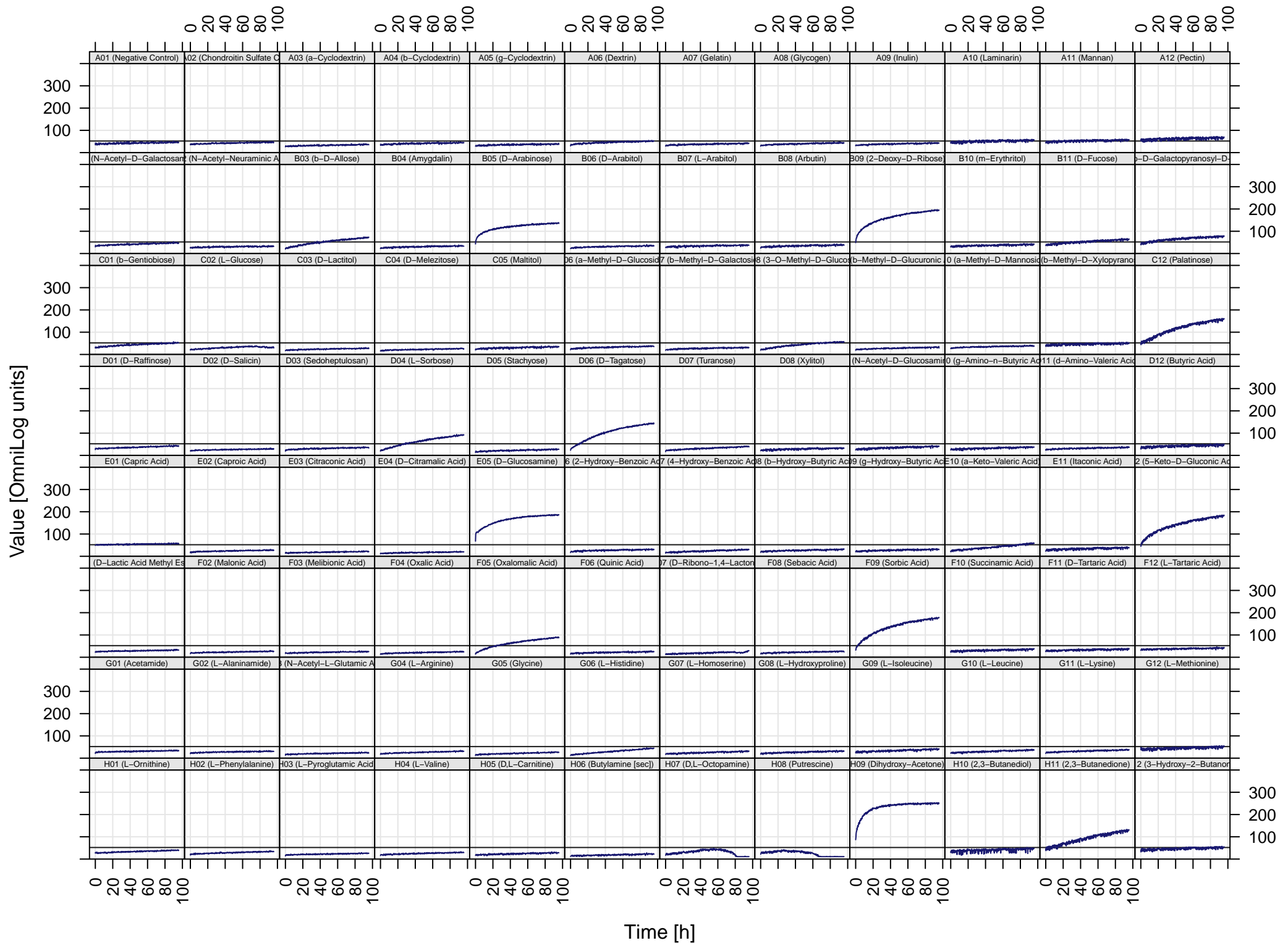

# PM03 (Nitrogen Sources)

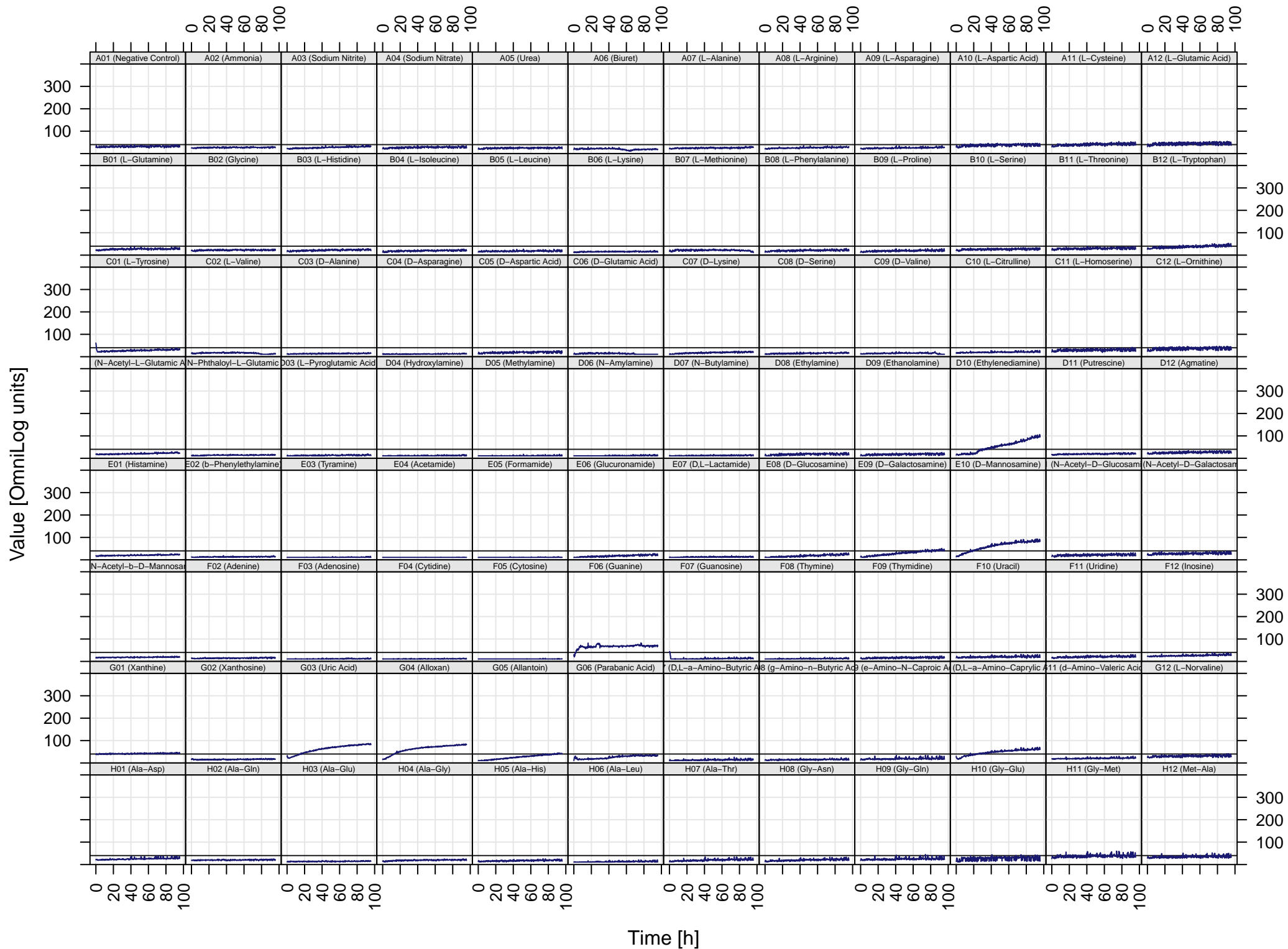

PM04 (Phosphorus and Sulfur Sources)

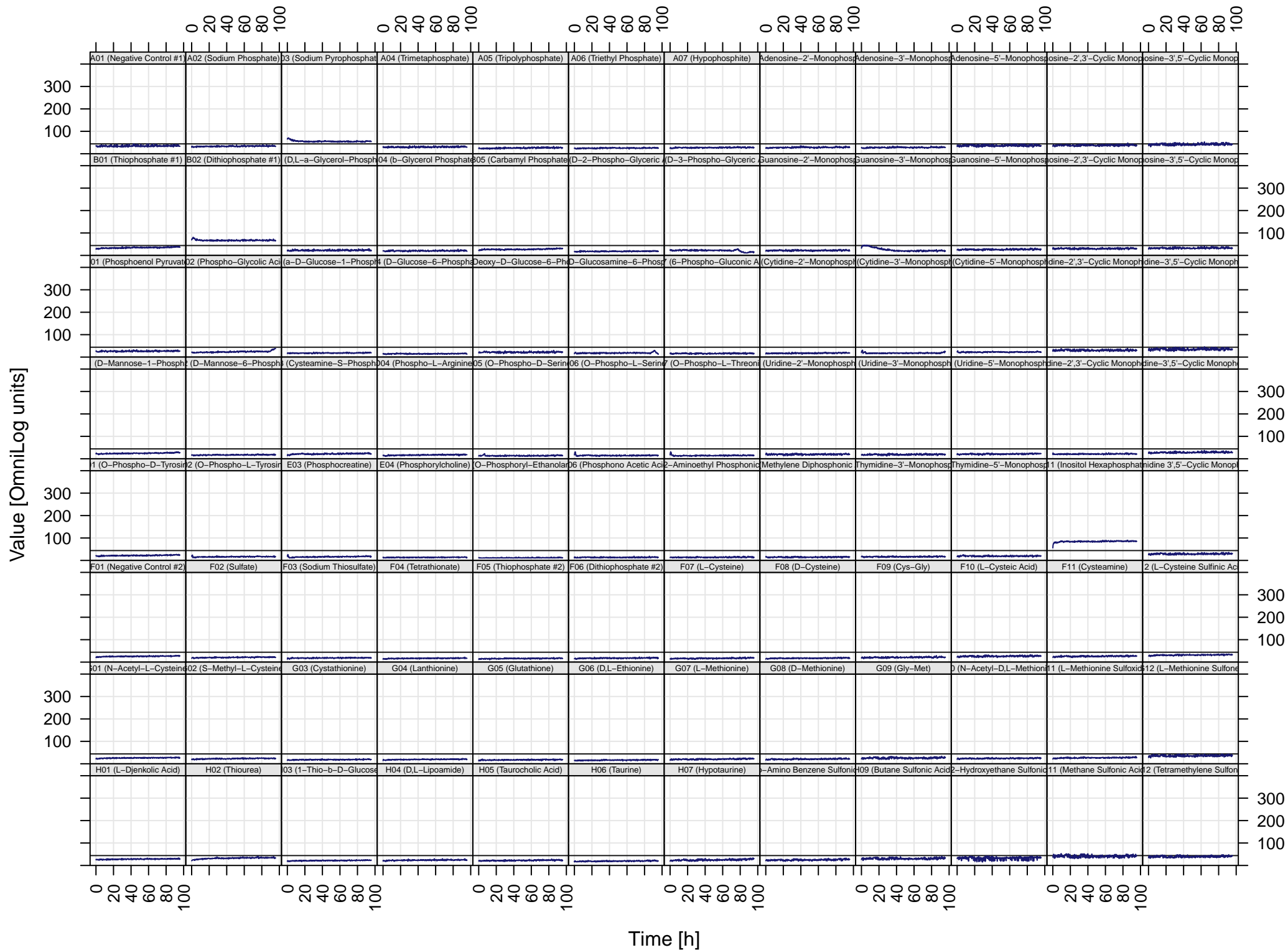

Supplement: Figure S1 — In the X-axis time is reported. In the Y-axis Omnilog units are reported. The Omnilog unit is a measurement of dye reduction and therefore a measurement of bacterial respiration. Wells in which the dye reduction is observed usually became positive soon and have a flat line over the time. [file peerj-05-2833-s001.pdf]

# PM01 (Carbon Sources)

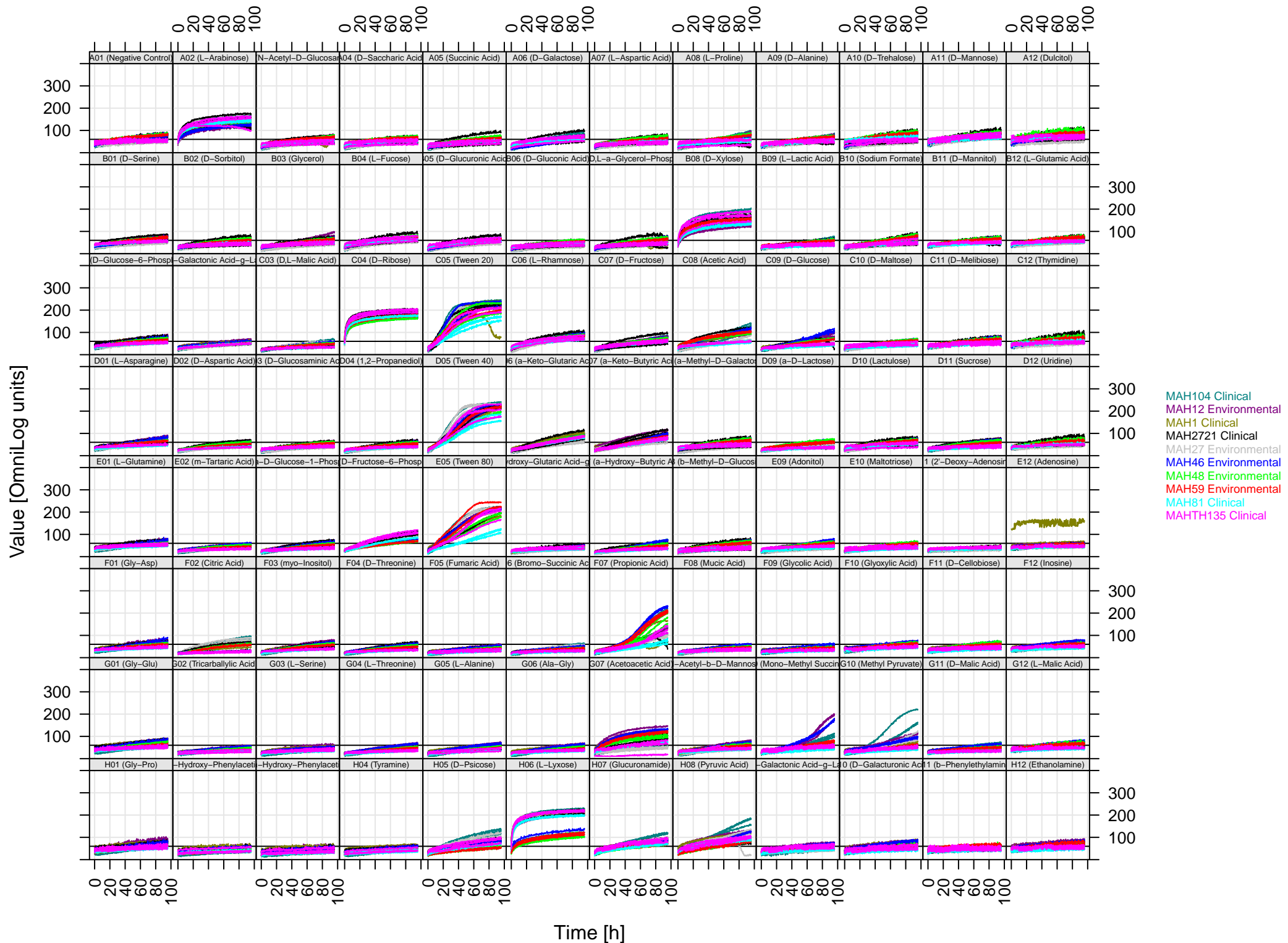

## PM02 (Carbon Sources)

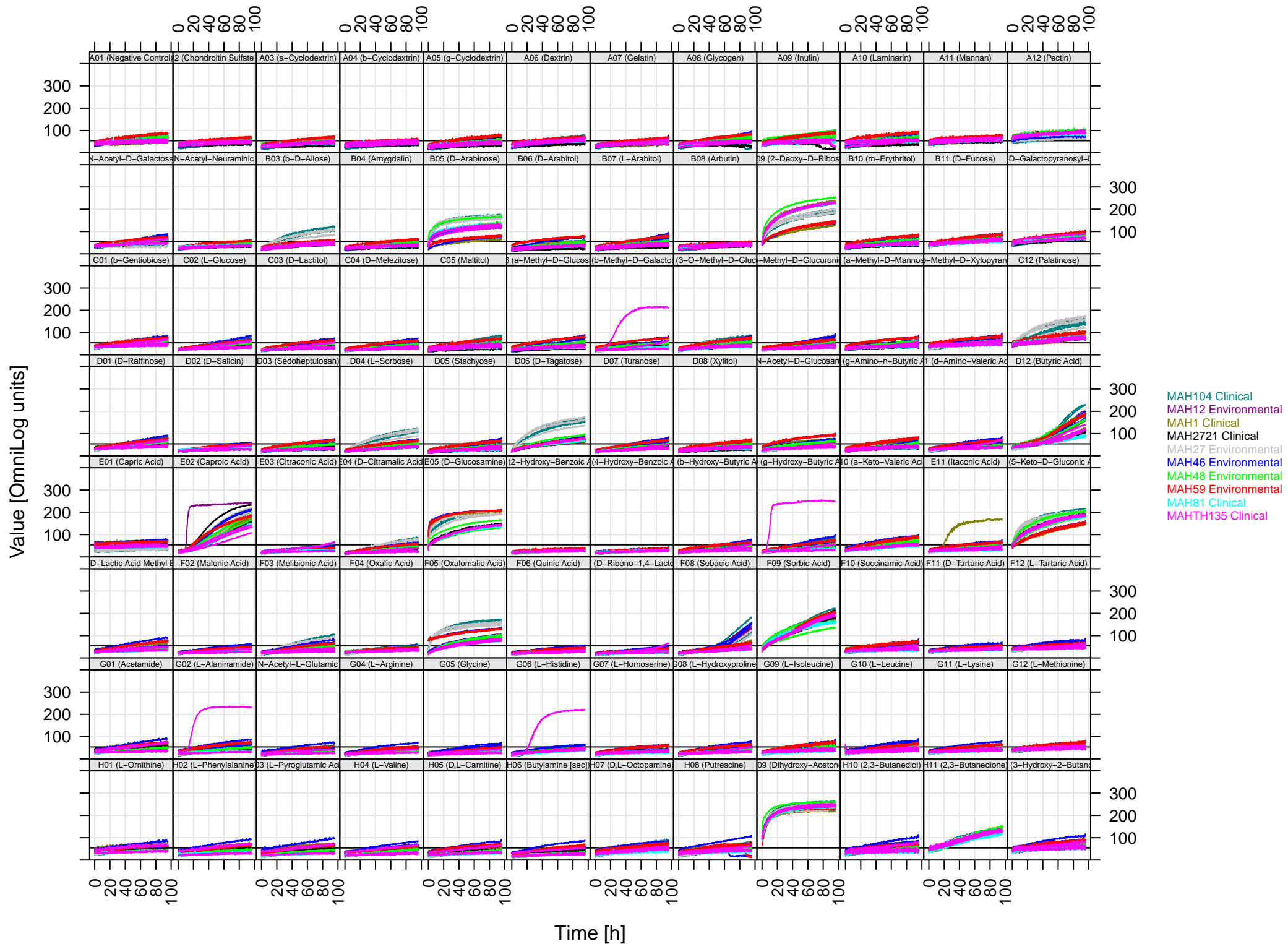

## PM03 (Nitrogen Sources)

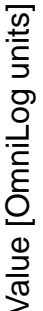

# PM04 (Phosphorus and Sulfur Sources)

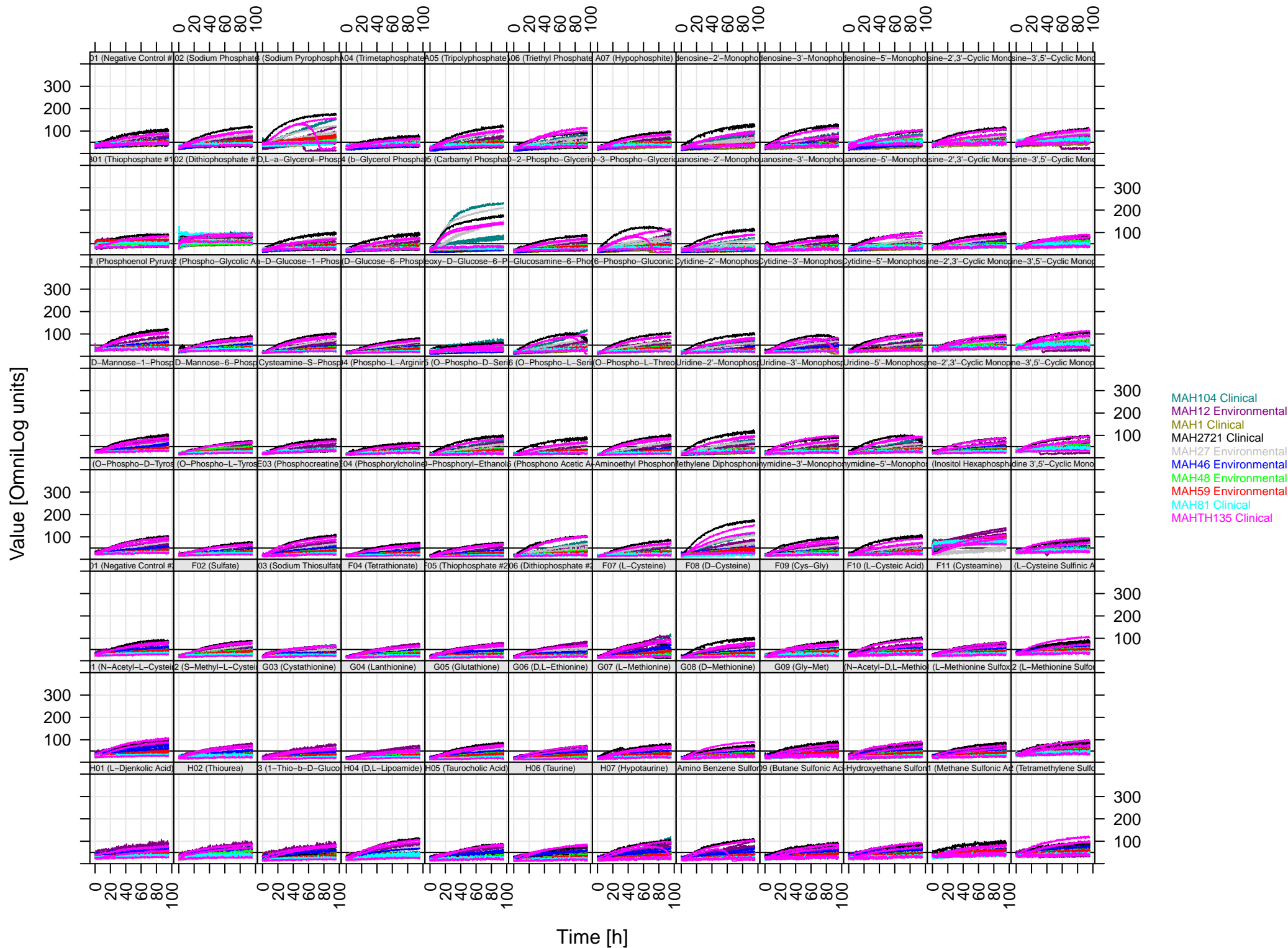

Supplement: Figure S2 — In the X-axis time is reported. In the Y-axis Omnilog units are reported. The Omnilog unit is a measurement of dye reduction and therefore a measurement of bacterial respiration. Each color represents a different isolate. For each isolate, the median value among the three replicates is given. [file peerj-05-2833-s002.pdf]
