# Supplementary material for: Metabolic phenotype of clinical and environmental Mycobacterium avium subsp. hominissuis isolates
Source: PeerJ. 2017 Jan 3;5:e2833. doi: 10.7717/peerj.2833 (PMC5214758; doi:10.7717/peerj.2833)
Supplement: Table S1 [file peerj-05-2833-s003.pdf]

Table S1. The 334 substrates showing negative reaction in all the ten MAH isolates tested, listed by PM plates.

PM1

Carbon substrates

1,"A03 (N-Acetyl-D-Glucosamine)"
2,"A04 (D-Saccharic Acid)"
3,"A05 (Succinic Acid)"
4,"A06 (D-Galactose)"
5,"A07 (L-Aspartic Acid)"
6,"A08 (L-Proline)"
7,"A09 (D-Alanine)"
8,"A10 (D-Trehalose)"
9,"A11 (D-Mannose)"
10,"A12 (Dulcitol)"
11,"B01 (D-Serine)"
12,"B02 (D-Sorbitol)"
13,"B03 (Glycerol)"
14,"B04 (L-Fucose)"
15,"B05 (D-Glucuronic Acid)"
16,"B06 (D-Gluconic Acid)"
17,"B07 (D,L-a-Glycerol-Phosphate)"
18,"B09 (L-Lactic Acid)"
19,"B10 (Sodium Formate)"
20,"B11 (D-Mannitol)"
21,"B12 (L-Glutamic Acid)"
22,"C01 (D-Glucose-6-Phosphate)"
23,"C02 (D-Galactonic Acid-g-Lactone)"
24,"C03 (D,L-Malic Acid)"
25,"C07 (D-Fructose)"
26,"C09 (D-Glucose)"
27,"C10 (D-Maltose)"
28,"C11 (D-Melibiose)"
29,"C12 (Thymidine)"
30,"D01 (L-Asparagine)"
31,"D02 (D-Aspartic Acid)"
32,"D03 (D-Glucosaminic Acid)"
33,"D04 (1,2-Propanediol)"
34,"D06 (a-Keto-Glutaric Acid)"
35,"D07 (a-Keto-Butyric Acid)"
36,"D08 (a-Methyl-D-Galactoside)"
37,"D09 (a-D-Lactose)"
38,"D10 (Lactulose)"
39,"D11 (Sucrose)"
40,"D12 (Uridine)"
41,"E01 (L-Glutamine)"
42,"E02 (m-Tartaric Acid)"
43,"E03 (a-D-Glucose-1-Phosphate)"
44,"E06 (a-Hydroxy-Glutaric Acid-g-Lactone)"

45,"E07 (a-Hydroxy-Butyric Acid)"
46,"E08 (b-Methyl-D-Glucoside)"
47,"E09 (Adonitol)"
48,"E10 (Maltotriose)"
49,"E11 (2'-Deoxy-Adenosine)"
50,"E12 (Adenosine)"
51,"F01 (Gly-Asp)"
52,"F02 (Citric Acid)"
53,"F03 (myo-Inositol)"
54,"F04 (D-Threonine)"
55,"F05 (Fumaric Acid)"
56,"F06 (Bromo-Succinic Acid)"
57,"F08 (Mucic Acid)"
58,"F09 (Glycolic Acid)"
59,"F10 (Glyoxylic Acid)"
60,"F11 (D-Cellobiose)"
61,"F12 (Inosine).x"
62,"G01 (Gly-Glu)"
63,"G02 (Tricarballylic Acid)"
64,"G03 (L-Serine)"
65,"G04 (L-Threonine)"
66,"G05 (L-Alanine)"
67,"G06 (Ala-Gly)"
68,"G08 (N-Acetyl-b-D-Mannosamine)"
69,"G11 (D-Malic Acid)"
70,"G12 (L-Malic Acid)"
71,"H01 (Gly-Pro)"
72,"H02 (p-Hydroxy-Phenylacetic Acid)"
73,"H03 (m-Hydroxy-Phenylacetic Acid)"
74,"H04 (Tyramine)"
75,"H07 (Glucuronamide)"
76,"H09 (L-Galactonic Acid-g-Lactone)"
77,"H10 (D-Galacturonic Acid)"
78,"H11 (b-Phenylethylamine)"
79,"H12 (Ethanolamine)"

PM2

Carbon substrates

80,"A02 (Chondroitin Sulfate C)"
81,"A03 (a-Cyclodextrin)"
82,"A04 (b-Cyclodextrin)"
83,"A05 (g-Cyclodextrin)"
84,"A06 (Dextrin)"
85,"A07 (Gelatin)"
86,"A08 (Glycogen)"
87,"A09 (Inulin)"
88,"A10 (Laminarin)"
89,"A11 (Mannan)"
90,"A12 (Pectin)"
91,"B01 (N-Acetyl-D-Galactosamine)"
92,"B02 (N-Acetyl-Neuraminic Acid)"

93,"B03 (b-D-Allose)"
94,"B04 (Amygdalin)"
95,"B06 (D-Arabitol)"
96,"B07 (L-Arabitol)"
97,"B08 (Arbutin)"
98,"B10 (m-Erythritol)"
99,"B11 (D-Fucose)"
100,"C01 (b-Gentiobiose)"
101,"C02 (L-Glucose)"
102,"C03 (D-Lactitol)"
103,"C04 (D-Melezitose)"
104,"C05 (Maltitol)"
105,"C06 (a-Methyl-D-Glucoside)"
106,"C08 (3-O-Methyl-D-Glucose)"
107,"C09 (b-Methyl-D-Glucuronic Acid)"
108,"C10 (a-Methyl-D-Mannoside)"
109,"C11 (b-Methyl-D-Xylopyranoside)"
110,"D01 (D-Raffinose)"
111,"D02 (D-Salicin)"
112,"D03 (Sedoheptulosan)"
113,"D05 (Stachyose)"
114,"D07 (Turanose)"
115,"D08 (Xylitol)"
116,"D09 (N-Acetyl-D-Glucosaminitol)"
117,"D10 (g-Amino-n-Butyric Acid)"
118,"D11 (d-Amino-Valeric Acid)"
119,"E01 (Capric Acid)"
120,"E03 (Citraconic Acid)"
121,"E04 (D-Citramalic Acid)"
122,"E06 (2-Hydroxy-Benzoic Acid)"
123,"E07 (4-Hydroxy-Benzoic Acid)"
124,"E08 (b-Hydroxy-Butyric Acid)"
125,"E10 (a-Keto-Valeric Acid)"
126,"E11 (Itaconic Acid)"
127,"F01 (D-Lactic Acid Methyl Ester)"
128,"F02 (Malonic Acid)"
129,"F03 (Melibionic Acid)"
130,"F04 (Oxalic Acid)"
131,"F06 (Quinic Acid)"
132,"F07 (D-Ribono-1,4-Lactone)"
133,"F10 (Succinamic Acid)"
134,"F11 (D-Tartaric Acid)"
135,"F12 (L-Tartaric Acid)"
136,"G01 (Acetamide)"
137,"G03 (N-Acetyl-L-Glutamic Acid)"
138,"G04 (L-Arginine)"
139,"G05 (Glycine)"
140,"G07 (L-Homoserine)"
141,"G08 (L-Hydroxyproline)"
142,"G09 (L-Isoleucine)"

143,"G10 (L-Leucine)"
144,"G11 (L-Lysine)"
145,"G12 (L-Methionine)"
146,"H01 (L-Ornithine)"
147,"H02 (L-Phenylalanine)"
148,"H03 (L-Pyroglutamic Acid)"
149,"H04 (L-Valine)"
150,"H05 (D,L-Carnitine)"
151,"H06 (Butylamine [sec])"
152,"H07 (D,L-Octopamine)"
153,"H08 (Putrescine)"
154,"H10 (2,3-Butanediol)"
155,"H12 (3-Hydroxy-2-Butanone)"

PM3

Nitrogen substrates

156,"A02 (Ammonia)"
157,"A03 (Sodium Nitrite)"
158,"A04 (Sodium Nitrate)"
159,"A05 (Urea)"
160,"A06 (Biuret)"
161,"A07 (L-Alanine)"
162,"A08 (L-Arginine)"
163,"A09 (L-Asparagine)"
164,"A10 (L-Aspartic Acid)"
165,"A12 (L-Glutamic Acid)"
166,"B01 (L-Glutamine)"
167,"B02 (Glycine)"
168,"B03 (L-Histidine)"
169,"B04 (L-Isoleucine)"
170,"B05 (L-Leucine)"
171,"B06 (L-Lysine)"
172,"B07 (L-Methionine)"
173,"B08 (L-Phenylalanine)"
174,"B09 (L-Proline)"
175,"B10 (L-Serine)"
176,"B11 (L-Threonine)"
177,"B12 (L-Tryptophan)"
178,"C01 (L-Tyrosine)"
179,"C02 (L-Valine)"
180,"C03 (D-Alanine)"
181,"C04 (D-Asparagine)"
182,"C05 (D-Aspartic Acid)"
183,"C06 (D-Glutamic Acid)"
184,"C07 (D-Lysine)"
185,"C08 (D-Serine)"
186,"C09 (D-Valine)"
187,"C10 (L-Citrulline)"
188,"C11 (L-Homoserine)"
189,"C12 (L-Ornithine)"
190,"D01 (N-Acetyl-L-Glutamic Acid)"

191,"D02 (N-Phthaloyl-L-Glutamic Acid)"
192,"D03 (L-Pyroglutamic Acid)"
193,"D04 (Hydroxylamine)"
194,"D05 (Methylamine)"
195,"D06 (N-Amylamine)"
196,"D07 (N-Butylamine)"
197,"D08 (Ethylamine)"
198,"D09 (Ethanolamine)"
199,"D11 (Putrescine)"
200,"D12 (Agmatine)"
201,"E01 (Histamine)"
202,"E02 (b-Phenylethylamine)"
203,"E03 (Tyramine)"
204,"E04 (Acetamide)"
205,"E05 (Formamide)"
206,"E06 (Glucuronamide)"
207,"E07 (D,L-Lactamide)"
208,"E08 (D-Glucosamine)"
209,"E11 (N-Acetyl-D-Glucosamine)"
210,"E12 (N-Acetyl-D-Galactosamine)"
211,"F01 (N-Acetyl-b-D-Mannosamine)"
212,"F02 (Adenine)"
213,"F03 (Adenosine)"
214,"F04 (Cytidine)"
215,"F05 (Cytosine)"
216,"F06 (Guanine)"
217,"F07 (Guanosine)"
218,"F08 (Thymine)"
219,"F09 (Thymidine)"
220,"F10 (Uracil)"
221,"F11 (Uridine)"
222,"F12 (Inosine).y"
223,"G01 (Xanthine)"
224,"G02 (Xanthosine)"
225,"G05 (Allantoin)"
226,"G06 (Parabanic Acid)"
227,"G07 (D,L-a-Amino-Butyric Acid)"
228,"G08 (g-Amino-n-Butyric Acid)"
229,"G09 (e-Amino-N-Caproic Acid)"
230,"G11 (d-Amino-Valeric Acid)"
231,"G12 (L-Norvaline)"
232,"H01 (Ala-Asp)"
233,"H02 (Ala-Gln)"
234,"H03 (Ala-Glu)"
235,"H04 (Ala-Gly)"
236,"H05 (Ala-His)"
237,"H06 (Ala-Leu)"
238,"H07 (Ala-Thr)"
239,"H08 (Gly-Asn)"
240,"H09 (Gly-Gln)"

241,"H10 (Gly-Glu)"
242,"H11 (Gly-Met)"
243,"H12 (Met-Ala)"
PM4
Phosphorous substrates
244,"A02 (Sodium Phosphate)"
245,"A04 (Trimetaphosphate)"
246,"A05 (Tripolyphosphate)"
247,"A06 (Triethyl Phosphate)"
248,"A07 (Hypophosphite)"
249,"A08 (Adenosine-2'-Monophosphate)"
250,"A09 (Adenosine-3'-Monophosphate)"
251,"A10 (Adenosine-5'-Monophosphate)"
252,"A11 (Adenosine-2',3'-Cyclic Monophosphate)"
253,"A12 (Adenosine-3',5'-Cyclic Monophosphate)"
254,"B01 (Thiophosphate #1)"
255,"B02 (Dithiophosphate #1)"
256,"B03 (D,L-a-Glycerol-Phosphate)"
257,"B04 (b-Glycerol Phosphate)"
258,"B06 (D-2-Phospho-Glyceric Acid)"
259,"B07 (D-3-Phospho-Glyceric Acid)"
260,"B08 (Guanosine-2'-Monophosphate)"
261,"B09 (Guanosine-3'-Monophosphate)"
262,"B10 (Guanosine-5'-Monophosphate)"
263,"B11 (Guanosine-2',3'-Cyclic Monophosphate)"
264,"B12 (Guanosine-3',5'-Cyclic Monophosphate)"
265,"C01 (Phosphoenol Pyruvate)"
266,"C02 (Phospho-Glycolic Acid)"
267,"C03 (a-D-Glucose-1-Phosphate)"
268,"C04 (D-Glucose-6-Phosphate)"
269,"C05 (2-Deoxy-D-Glucose-6-Phosphate)"
270,"C06 (D-Glucosamine-6-Phosphate)"
271,"C07 (6-Phospho-Gluconic Acid)"
272,"C08 (Cytidine-2'-Monophosphate)"
273,"C09 (Cytidine-3'-Monophosphate)"
274,"C10 (Cytidine-5'-Monophosphate)"
275,"C11 (Cytidine-2',3'-Cyclic Monophosphate)"
276,"C12 (Cytidine-3',5'-Cyclic Monophosphate)"
277,"D01 (D-Mannose-1-Phosphate)"
278,"D02 (D-Mannose-6-Phosphate)"
279,"D03 (Cysteamine-S-Phosphate)"
280,"D04 (Phospho-L-Arginine)"
281,"D05 (O-Phospho-D-Serine)"
282,"D06 (O-Phospho-L-Serine)"
283,"D07 (O-Phospho-L-Threonine)"
284,"D08 (Uridine-2'-Monophosphate)"
285,"D09 (Uridine-3'-Monophosphate)"
286,"D10 (Uridine-5'-Monophosphate)"
287,"D11 (Uridine-2',3'-Cyclic Monophosphate)"
288,"D12 (Uridine-3',5'-Cyclic Monophosphate)"

289,"E01 (O-Phospho-D-Tyrosine)"
290,"E02 (O-Phospho-L-Tyrosine)"
291,"E03 (Phosphocreatine)"
292,"E04 (Phosphorylcholine)"
293,"E05 (O-Phosphoryl-Ethanolamine)"
294,"E06 (Phosphono Acetic Acid)"
295,"E07 (2-Aminoethyl Phosphonic Acid)"
296,"E08 (Methylene Diphosphonic Acid)"
297,"E09 (Thymidine-3'-Monophosphate)"
298,"E10 (Thymidine-5'-Monophosphate)"
299,"E12 (Thymidine 3',5'-Cyclic Monophosphate)"
Sulfur substrates
300,"F02 (Sulfate)"
301,"F03 (Sodium Thiosulfate)"
302,"F04 (Tetrathionate)"
303,"F05 (Thiophosphate #2)"
304,"F06 (Dithiophosphate #2)"
305,"F07 (L-Cysteine)"
306,"F08 (D-Cysteine)"
307,"F09 (Cys-Gly)"
308,"F10 (L-Cysteic Acid)"
309,"F11 (Cysteamine)"
310,"F12 (L-Cysteine Sulfinic Acid)"
311,"G01 (N-Acetyl-L-Cysteine)"
312,"G02 (S-Methyl-L-Cysteine)"
313,"G03 (Cystathionine)"
314,"G04 (Lanthionine)"
315,"G05 (Glutathione)"
316,"G06 (D,L-Ethionine)"
317,"G07 (L-Methionine)"
318,"G08 (D-Methionine)"
319,"G09 (Gly-Met)"
320,"G10 (N-Acetyl-D,L-Methionine)"
321,"G11 (L-Methionine Sulfoxide)"
322,"G12 (L-Methionine Sulfone)"
323,"H01 (L-Djenkolic Acid)"
324,"H02 (Thiourea)"
325,"H03 (1-Thio-b-D-Glucose)"
326,"H04 (D,L-Lipoamide)"
327,"H05 (Taurocholic Acid)"
328,"H06 (Taurine)"
329,"H07 (Hypotaurine)"
330,"H08 (p-Amino Benzene Sulfonic Acid)"
331,"H09 (Butane Sulfonic Acid)"
332,"H10 (2-Hydroxyethane Sulfonic Acid)"
333,"H11 (Methane Sulfonic Acid)"
334,"H12 (Tetramethylene Sulfone)"
