# Supplementary material for: Metabolic phenotype of clinical and environmental Mycobacterium avium subsp. hominissuis isolates
Source: PeerJ. 2017 Jan 3;5:e2833. doi: 10.7717/peerj.2833 (PMC5214758; doi:10.7717/peerj.2833)
Supplement: Table S2 — The number and letter of each substrate indicate the exact position in the PM plate. [file peerj-05-2833-s004.pdf]

Table S2. List of the 23 wells causing abiotic dye reduction. The number and letter of each substrate indicate the exact position in the PM plate.

	Plates			
	**PM1**	**PM2**	**PM3**	**PM4**
**Substrates**	A-02 L-arabinose	B-05 D-arabinose	D-10 Ethylenediamine	E-11 Inositol Exaphosphate
	B-08 D-xylose	B-09 2 deoxy-D-ribose	E-10 D-mannosamine	
	C-04 D-ribose	B-12 3-0-β-D-galactopyranosyl-D-arabinose	G-03 uric acid	
	C-06 L-rhamnose	C-12 palatinose	G-04 Alloxan	
	E-04 D-fructose-6-phospate	D-04 L-sorbose		
	H-06 L-lyxose	D-06 D-tagatose		
		E-05 D-glucosamine		
		E-12, 5-keto-D-gluconic acid		
		F-05 oxalomalic acid		
		F-09 sorbic acid,		
		H-09 dihydroxyacetone		
		H-11 2,3 butanedione		
